# E-cadherin transcriptional downregulation by promoter methylation but not mutation is related to epithelial-to-mesenchymal transition in breast cancer cell lines

**DOI:** 10.1038/sj.bjc.6602996

**Published:** 2006-02-21

**Authors:** M Lombaerts, T van Wezel, K Philippo, J W F Dierssen, R M E Zimmerman, J Oosting, R van Eijk, P H Eilers, B van de Water, C J Cornelisse, A-M Cleton-Jansen

**Affiliations:** 1 Department of Pathology, Leiden University Medical Center, PO Box 9600, L1-Q, NL-2300 RC Leiden, The Netherlands; 2 Medical Statistics, Leiden University Medical Center, PO Box 9604, NL-2300 RC Leiden, The Netherlands; 3 Division of Toxicology, Leiden/Amsterdam Center for Drug Research, Leiden University, PO Box 9502, NL-2300 RA Leiden, The Netherlands

**Keywords:** E-cadherin, epithelial–mesenchymal transition, promoter methylation, mammary cell lines, TFG*β* pathway

## Abstract

Using genome-wide expression profiling of a panel of 27 human mammary cell lines with different mechanisms of E-cadherin inactivation, we evaluated the relationship between E-cadherin status and gene expression levels. Expression profiles of cell lines with E-cadherin (*CDH1*) promoter methylation were significantly different from those with *CDH1* expression or, surprisingly, those with *CDH1* truncating mutations. Furthermore, we found no significant differentially expressed genes between cell lines with wild-type and mutated *CDH1*. The expression profile complied with the fibroblastic morphology of the cell lines with promoter methylation, suggestive of epithelial–mesenchymal transition (EMT). All other lines, also the cases with *CDH1* mutations, had epithelial features. Three non-tumorigenic mammary cell lines derived from normal breast epithelium also showed *CDH1* promoter methylation, a fibroblastic phenotype and expression profile. We suggest that *CDH1* promoter methylation, but not mutational inactivation, is part of an entire programme, resulting in EMT and increased invasiveness in breast cancer. The molecular events that are part of this programme can be inferred from the differentially expressed genes and include genes from the TGF*β* pathway, transcription factors involved in *CDH1* regulation (i.e. ZFHX1B, SNAI2, but not SNAI1, TWIST), annexins, AP1/2 transcription factors and members of the actin and intermediate filament cytoskeleton organisation.

Decreased E-cadherin expression is associated with a more aggressive behaviour of breast cancer (Siitonen *et al*, 1996). The homotypic cellular adhesion molecule E-cadherin is a transmembrane glycoprotein important for the organisation of epithelial structure (Takeichi, 1995; Huber *et al*, 1996; Yagi and Takeichi, 2000). E-cadherin can form homophilic interactions with E-cadherin molecules on neighbouring cells in a Ca^2+^-dependent way and is the main component of adherens junctions. By recruitment of *α*- and *β*-catenin, the E-cadherin is anchored to the actin cytoskeleton.

Mutational inactivation of *CDH1* has been found in 56% of lobular breast carcinomas (Berx *et al*, 1996) and 50% of diffuse gastric carcinomas (Becker *et al*, 1994). In the first tumour type, this is accompanied by loss of the wild-type allele (Berx *et al*, 1995). Complete loss of E-cadherin protein expression has been found in 84% of lobular breast carcinomas (De Leeuw *et al*, 1997). Loss of membranous E-cadherin expression results in a reduction of adhesion between epithelial tumour cells and explains the characteristic diffuse growth pattern observed in these tumours (Berx *et al*, 1998). *CDH1* mutations were also identified in lobular carcinoma *in situ*, the putative precursor of invasive lobular carcinoma (Vos *et al*, 1997). Thus, in addition to its role as an invasion suppressor, E-cadherin also acts as a classical tumour suppressor gene in pre-invasive lobular breast carcinoma. Besides mutational inactivation of the E-cadherin gene, *CDH1* may also be targeted by promoter hypermethylation (Graff *et al*, 1995; Grady *et al*, 2000; Tamura *et al*, 2000), thereby inhibiting *CDH1* gene expression. Evidence is accumulating for a prominent role of epithelial-to-mesenchymal transition (EMT) in tumour progression (reviewed by Thiery, 2002). During early embryonic development, E-cadherin is a critical switch in EMT. Upon downregulation of E-cadherin, epithelial cells acquire a fibroblastic phenotype, dissociate from the epithelium and migrate. This process is essential for gastrulation, neural crest formation, kidney development and so on (reviewed by Thiery, 2003). Several proteins have been identified that downregulate E-cadherin expression including SNAI1/SNAIL (Batlle *et al*, 2000; Cano *et al*, 2000), ZFHX1B/SIP1 (Comijn *et al*, 2001), SNAI2/SLUG (Hajra *et al*, 2002; De Craene *et al*, 2005), TWIST1 (Yang *et al*, 2004) and DeltaEF1 (Eger *et al*, 2005). Altered expression of these transcription factors seems to be also associated with an altered overexpression of transcriptional repressors of E-cadherin in tumour cells (Batlle *et al*, 2000; Cano *et al*, 2000; Comijn *et al*, 2001; Hajra *et al*, 2002; Yang *et al*, 2004; Eger *et al*, 2005). *CDH1* gene expression is upregulated by several factors, AML1, p300 and HNF3 (Liu *et al*, 2005). Also post-transcriptional regulation of E-cadherin has been observed and recently ADAM10 was identified as the cleaving protease (Maretzky *et al*, 2005).

Given the relevance of E-cadherin in tumour development and progression and the different mechanisms involved in its regulation, we set out to use a genome-wide expression analysis to identify genes or pathways in mammary epithelial cells that are either (in)directly affected by loss of E-cadherin function or are altogether associated with a (epi)genetic programme that determines the biological status of cells: epithelial or mesenchymal. For this purpose, we performed a gene expression profile study on 27 different breast mammary cell lines with a known *CDH1* mutation, promoter hypermethylation and expression status. Remarkably, no significant differences in gene expression were identified between breast cancer cell lines with wild-type E-cadherin without promoter methylation and those harbouring *CDH1* truncating mutations. The results showed a marked difference in expression profile between cell lines with *CDH1* promoter methylation compared to those with *CDH1* mutational inactivation, especially for genes involved in EMT and part of the TGF*β* pathway.

## MATERIALS AND METHODS

### Cell lines

The 27 breast and four colon cancer cell lines used in this study are listed in Table 1. MPE600 and SK-BR-5 were provided by Dr F Waldman (California Pacific Medical Centre, San Francisco, CA, USA) and Dr E Stockert (Sloan-Kettering Institute for Cancer Research, New York, NY, USA), respectively. Dr SP Ethier donated SUM44PE (Ethier *et al*, 1993) and SUM185PE (Forozan *et al*, 1999). OCUB-F was obtained from the Riken Gene Bank. Other cell lines were obtained from the American Type Culture Collection. We genotyped all cell lines using the Powerplex 1.2 system (Promega, Leiden, The Netherlands) according to the manufacturer's instructions. All cell lines were grown in RPMI1640 medium supplemented with 5 mM glutamine and 10% heat-inactivated fetal calf serum at 37°C under 5% CO_2_ (culture media from GIBCO Invitrogen, Grand Island, NY, USA).

### E-cadherin protein expression

Cells were grown until 80% confluence and lysed by adding 1 ml hot lysis solution (1% SDS, 10 mM Tris pH 7.4, 10 mM EDTA, supplemented with complete protease inhibitor (Boehringer Mannheim)). Protein concentration was determined by the Biorad DC Protein Assay (Biorad, Hercules, CA, USA). Western blots of electrophoretically separated proteins (Winter *et al*, 2003) from whole-cell lysates were probed with HECD-1 (Zymed Laboratories, San Francisco, CA, USA) antibodies to detect E-cadherin (diluted 1 : 1000). Control blots were probed with anti-*β*-actin (1 : 500) (Sigma, St Louis, MO, USA). Anti-mouse IgG peroxidase conjugates (Transduction Laboratories, Lexington, KY, USA) were used as secondary antibodies, and the blots were developed for 1 min using the enhanced chemiluminescent detection system (Amersham Int., Little Chalfont, UK). Exposure time was 1 min. Membranous E-cadherin expression was analysed using HECD-1 by flow cytometry of viable cells as described previously (Corver *et al*, 1994). In brief, 1 *μ*g of HECD1 was used to label the cells. For each sample, measurements from 20 000 single cells were collected using a standard FACSCalibur™ flow cytometer (BD Biosciences, San Diego, CA, USA). Data were analysed using WinList 5.0 software (Verity Software House Inc., Topsham, ME, USA).

### Methylation-specific PCR

*CDH1* promoter methylation was determined as described by Herman *et al* (1996). Modification of DNA before PCR was carried out with the EZ Methylation Kit™ (Zymo Research, Orange, CA, USA) according to the manufacturer's instructions. Methylation-specific fragments were sequenced to determine methylation of CpGs between the primer binding sites. As a control for efficient modification, we used primers for fragments of *CDH3* and *TERF2*, flanking *CDH1*, which do not contain CpGs and exclusively amplified modified DNA (primer sequences and PCR conditions are available upon request).

### RNA isolation

When cell cultures reached 70–80% confluence, RNA was isolated using TRIzol (Invitrogen Life Technologies Breda, The Netherlands) and purified using Qiagen RNeasy mini kit columns (Qiagen Sciences, Germantown, MD, USA). Samples were DNase treated using the Qiagen RNase-free DNase kit (Qiagen). The isolation and purification were carried out according to the manufacturer's instructions.

### cDNA microarrays

cDNA clones were amplified to generate PCR products for the cDNA microarray from a sequence verified clone collection (Research Genetics, Invitrogen, Huntsville, AL, USA). Apart from these clones, cDNAs related to adhesion, cytoskeleton and carcinogenesis were selected. These additional clones were obtained from the Deutsches Krebsforschungszentrum (Deutsches Ressourcenzentrum für Genomforschung, Berlin, Germany) or created by performing an RT–PCR (reverse transcriptase) reaction on RNA from mammary cell lines. Subsequently, these PCR products were cloned using the TOPO TA cloning kit (Invitrogen, Carlsbad, CA, USA). All plasmid inserts were PCR amplified using plasmid-specific primers.

Purified PCR products, in 3 × SSC buffer, were spotted in duplicate on MicroMax SuperChip™ I slides (Perkin-Elmer Life Science, Boston, MA, USA) at the Leiden Genome Technology Center (http://www.lgtc.nl/) using the OmniGrid 100 robot (GeneMachines, San Carlos, CA, USA). In total, 9216 PCR products, including ‘landing marks’, consisting of biotin- and fluorescein-labelled PCR products to facilitate spot identification, were spotted in duplicate on each slide.

### cDNA labelling and hybridisation

For cDNA labelling and signal amplification, we used the tyramide signal amplification (TSA) kit (Perkin-Elmer Life Science, Boston, MA, USA) according to the manufacturer's instructions with minor modification. A 1 *μ*g portion of RNA was used to generate biotin- or fluorescein-labelled cDNA. In general, cell line cDNA was labelled with fluorescein (Cy3) whereas reference cDNA was labelled with biotin (Cy5). This reference RNA consists of RNA isolated from several human tumour cell lines (HL-60, K562, NCI-H226, COLO205, SNB-19, LOX-IMVI, OVCAR-3, OVCAR-4, CAK-IPC-3, MCF7, Hs578 T, MCF10F, MCF12A, OUMS27 and SW1353), analogous to the panel described by Ross *et al* (2000). Labelled cDNA was purified on YM-30 Microcon columns (Millipore Corporation, Bedford, MA, USA), dissolved in the hybridisation buffer and applied to cDNA arrays. Slides were hybridised overnight at 65°C in Corning Hybridization Chambers (Corning, NY, USA). The TSA reaction and washing of the slides was carried out using ThermoShandon Coverplates (http://www.thermo.com/).

### Data analysis

Microarrays were scanned using the GeneTAC LSIV laser scanner (Genomic Solutions, Ann Arbor, MI, USA). Each slide was scanned at two different gain settings, namely, a low gain to avoid detector saturation by high-amplitude signals and high gain to improve signal detection from weakly expressed genes. This approach provides a larger dynamic range of signal detection. Fluorescent spots were detected and quantified using GenePix Pro 3.0 software (Axon Instruments Inc., Union City, CA, USA). An MS-Excel macro was created for automated spot selection. This enables exclusion of saturated spots and spots with a signal below the threshold. The remaining spots were corrected for local background noise. The intensity for both dyes of each spot was normalised to the median of all spots on the array. For each spot, the ratio of the sample to the reference was calculated. For spots measured both at high gain and low gain, ratios were averaged. Finally, ratios were log 10-transformed.

Unsupervised cluster analysis (using the options ‘Complete linkage’ and ‘Correlation’) was made on log 10-transformed ratios with Cluster 2.12 (Eisen *et al*, 1998) and visualised using Treeview 1.6 (MB Eisen, http://rana.lbl.gov/index.htm). For the identification of differentially expressed genes, R version 1.9.0 (http://www.R-project.org/) (R Development Core Team, 2004) using the Limma (linear models for microarray data) package of Bioconductor (http://www.bioconductor.org/) was applied. Limma is a moderated T-statistic that detects differentially expressed genes between groups given the natural variance within these groups, corrected for false discovery rate due to multiple testing (Wettenhall and Smyth, 2004). Cluster analysis was made for genes yielding a signal in at least 90% of the samples. Independent hybridisations including dye-swaps of the same sample generally clustered together; therefore, we averaged experimental data for every cell line. Furthermore, we averaged ratios of duplicate spots on the array to improve significance. Differentially expressed genes were determined for cDNAs that gave a signal in at least 80% of the samples.

To test reproducibility of hybridisation and data acquisition, duplicate or triplicate microarray hybridisations were performed for 13 of the 31 cell lines, and seven dye-swap experiments. As the results of the duplicate or triplicate array hybridisations of the same cell line are highly similar, these are averaged as well as duplicate spots.

### Real-time PCR

Quantitative real-time PCR (qPCR) was performed to verify results found by the cDNA microarray analysis and examine the expression profile of candidate genes (Table 4). Primers were developed with the Beacon Designer 3 software (Premier Biosoft International, Palo Alto, CA, USA). Primer sequences and PCR conditions are available upon request. Reactions were performed using qPCR Corekits for SybrGreen or TAQman probes (Eurogentec, Seraing, Belgium). Cycle threshold (*C*_t_) and starting quantities (SQ) were determined using the Biorad iCycler software (Biorad, Hercules, CA, USA). *C*_t_ and SQ values were normalised to the expression levels of three housekeeping genes, *HNRPM, CPSF6* and *TBP*, selected from the microarray results as being stably expressed (Van Wezel *et al*, 2005), using the geNorm program (Vandesompele *et al*, 2002). Statistical analysis (ANOVA) was carried out using SPSS 10.0 (SPSS Inc., Chicago, IL, USA).

## RESULTS

### Cell line characterisation

All breast cell lines were genotyped to verify their identity. For 18 cell lines, results could be verified by data available from the ATCC (http://www.atcc.org). Only for CAMA1, we were unable to identify the 9.3 allele of THO1 on chromosome 11p15.5. Although for six cell lines no genotypes of the loci tested with the Powerplex kit were available, all had a unique profile. The MDA-MB-231^*^ cell line is a derivative of MDA-MB-231 that spontaneously arose in our laboratory. Both genotypes are identical except the loss of allele 8 of TPOX in MDA-MD-231^*^. Mutations in the DNA sequence of the *CDH1* gene have been reported previously (van de Wetering *et al*, 2001).

**Figure 1:**
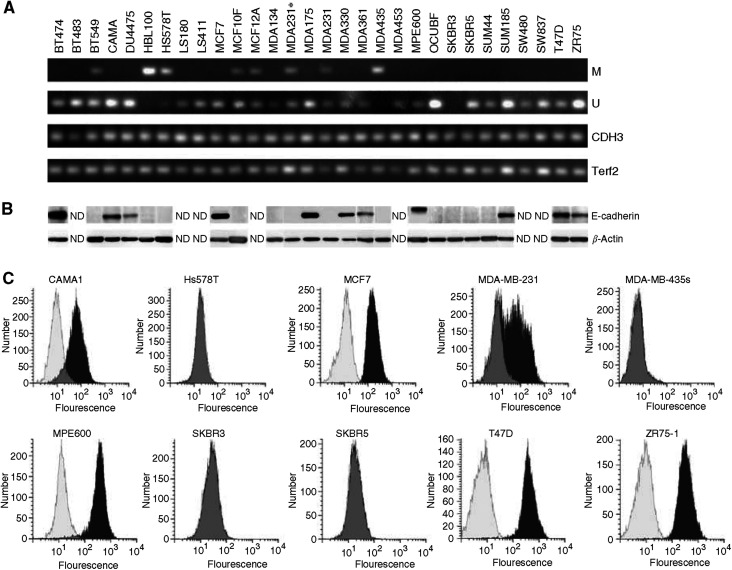
Verification of *CDH1* status in mammary cell lines. (**A**) Methylation-specific PCR. M=MSP specific for methylated CDH1 promoter, U=MSP specific for unmethylated CDH1 promoter, CDH3 and Terf2=control PCR fragments for integrity and modification of template DNA. (**B**) Western blot analysis for E-cadherin protein expression; *β*-actin is a loading control. (**C**) Fluorescence-activated cell sorting analysis for E-cadherin protein expression: overlay of control (white), without antibody and test (black), grey indicates overlap between control and test. The *y*-axis shows the number of cells and the *x*-axis the fluorescence of cells.

*CDH1* promoter methylation status was verified by methylation-specific PCR (MSP) (Figure 1A) (Graff *et al*, 1995; Herman *et al*, 1996; Hiraguri *et al*, 1998; Paz *et al*, 2003). Three different patterns were identified: (i) Complete promoter hypermethylation (cell lines HBL100 and MDA-MB-435s); (ii) partial promoter methylation (cell lines BT549, Hs578T, MDA-MB-231, MDA-MB-231^*^, MCF10F and MCF12A) and (iii) no promoter methylation in all other cell lines. Partial methylation indicates that not all CpGs in a promoter region are methylated, as reported previously (Lombaerts *et al*, 2004). This has been established by sequencing of the PCR products. The results are in agreement with published data for cell lines showing either complete or no promoter methylation. Cell lines showing partial promoter methylation (BT549 and MDA-MB-231) were previously reported as ‘methylated’ (Graff *et al*, 1995; Herman *et al*, 1996; Paz *et al*, 2003) without further specification. The partial *CDH1* promoter methylation of MCF10F and MCF12A was not published previously.

Western blotting of lysates of 28 cell lines confirmed the published presence or absence of E-cadherin protein expression (Sommers *et al*, 1994b; Hiraguri *et al*, 1998; Oberst *et al*, 2001; van de Wetering *et al*, 2001) (Figure 1B and Table 1).

Using flow cytometry on a subset of 11 cell lines, we detected membranous E-cadherin expression in five cell lines whereas six cell lines were negative (Figure 1C and Table 1). Highest expression was found for ZR75-1, MPE600 and T47D. These results were in agreement with those from Western blotting. The MDA-MB-231 cell line showed no E-cadherin expression by Western blotting (Figure 1B); however, the flow cytometric results on MDA-MB-231 indicated the presence of two subpopulations, one lacking E-cadherin protein and a weakly positive one (MESF value 18) (Figure 1C), apparently too weak to be detected by Western blot. Methylation-specific PCR analysis suggested partial promoter methylation (Figure 1A). Possibly, promoter methylation in this cell line is dynamic and reversible. This is illustrated by the lower mRNA level in a derivative of this cell line, MDA-MB-231^*^ (Table 4), and it is corroborated by recent findings (Kang *et al*, 2003) where subpopulations were selected from MDA-MB-231 cells with different metastatic propensities. This plasticity of the *CDH1* promoter has been observed previously (Graff *et al*, 2000).

The results on methylation status were in agreement with the protein expression data. Cell lines showing partial or complete promoter methylation lacked E-cadherin protein expression, with the exception of MDA-MB-231. Although the methylation profile was similar to that of other cell lines showing partial methylation (BT549, MCF10F and MCF12A), flow cytometry showed weak membranous protein expression in a subpopulation of the cells. For SK-BR-3, we could not detect a fragment in the MSP analysis. In order to verify the quality of DNA modification, we performed a PCR on modified DNA with primers specific for two fragments flanking *CDH1*. As PCR fragments were obtained for both flanking genes, a homozygous deletion of the DNA containing the *CDH1* promoter is likely. This result is in discordance with the reported loss of only exons 2–12 (van de Wetering *et al*, 2001).

### Cluster analysis

**Figure 2:**
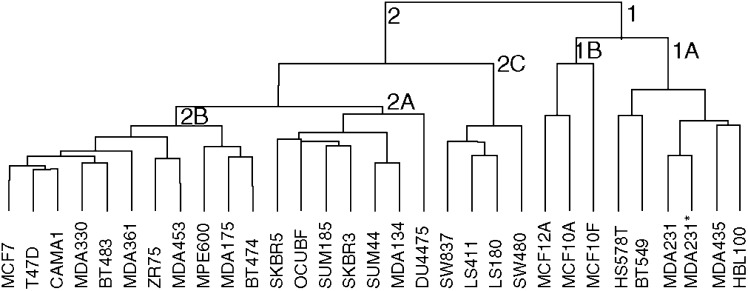
Hierarchical cluster analysis of cDNA microarray data for all cell lines reveals an ‘epithelial’ cluster (2) with wild-type *CDH1* (2A), mutated *CDH1* (2B) and colorectal cell lines (2C) and a ‘fibroblastic’ cluster (1), including tumour (1A) and ‘normal’ mammary cell lines (1B).

**Figure 3:**
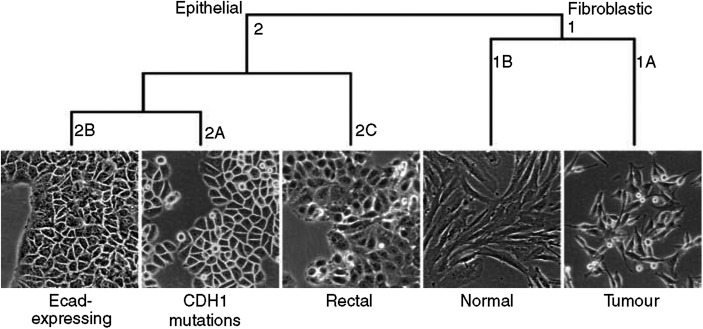
Morphology of representative cell lines in the different clusters. Cluster 1A is represented by MDA-MB-435, 1B by MCF10A, 2A by SKBR3, 2B by MCF7 and 2C by SW480.

Unsupervised cluster analysis of all cell lines identified two main clusters (Figure 2). Cluster 1 contains cell lines with a fibroblastic morphology, whereas the cluster 2 includes cell lines with a more or less epithelial appearance (Figure 3). The ‘Fibroblastic’ cluster includes two subclusters: 1A includes the breast cancer cell lines BT549, HBL100, Hs578T, MDA-MB-231, MDA-MB-231^*^ and MDA-MB-435s, which all show *CDH1* promoter methylation and are oestrogen receptor negative (ER^−^). Remarkably, cluster 1B (‘Fibroblastic-Normal’) contains three cell lines derived from normal breast tissue (MCF10A, MCF10F and MCF12A). Cluster 2, containing cell lines with an epithelial morphology, is divided into three subclusters. 2A (‘Epithelial-Ecad-expressing’) contains the cell lines BT483, BT474, MCF7, MDA-MB-175VII, MDA-MB-330, MDA-MB-361, MDA-MB-453, T47D and ZR75-1, with wild type *CDH1* and two cell lines with *CDH1* mutations, CAMA1 and MPE600. Cluster 2A includes eight ER^+^ cell lines and two ER^−^ cell lines. Interestingly, CAMA1 and MPE600 carry in-frame *CDH1* exon deletions (van de Wetering *et al*, 2001) and show membrane-bound E-cadherin protein expression in our flow cytometry analysis (Figure 1C). Thus, all cell lines in this cluster express E-cadherin protein, but interestingly, the size of the altered protein of MPE600 is larger than normal E-cadherin, suggesting a failure in the removal of the signal peptide. Cluster 2B (‘Epithelial-*CDH1*-mutated’) includes all breast cancer cell lines harbouring inactivating *CDH1* mutations (MDA-MB-134VI, SK-BR-3, SK-BR-5, SUM44PE and OCUB-F) and two cell lines with wild-type *CDH1*, SUM185PE and Du4475. 2B includes two ER^−^ and one ER^+^ cell line. Both Ocub-F and Du4475 grow in suspension, but no deviating growth pattern was observed for SUM185PE. Cluster 2C (‘Epithelial-Rectal’) contains cell lines derived from (colo-) rectal tumours (LS180, LS411N, SW480 and SW837). The separation of the colorectal cell lines validates the resolving power of the microarray method.

### Differentially expressed genes

To identify differentially expressed genes associated with differences in E-cadherin expression, we first compared the seven breast tumour cell lines in the ‘Epithelial’ cluster with *CDH1* mutations with 12 harbouring wild-type *CDH1* from the same cluster. Remarkably, we did not identify any significant differentially expressed genes using the Limma package. As CAMA1 and MPE600 harbour a mutation in the *CDH1* gene but still express E-cadherin, we next removed CAMA1 and MPE600 from this analysis. Also, this comparison did not yield any differentially expressed genes (data not shown).

Next, we compared the 18 breast tumour cell lines from the ‘Epithelial’ cluster with those from the ‘Fibroblastic’ cluster. We identified 121 clones showing a highly significant difference in expression (false discovery rate (FDR) <0.01), whereas an additional 187 clones showed differential expression at a lower level of significance (0.01⩽FDR<0.05). Twenty-eight genes were represented by two or more clones in this list. In total, we identified 273 genes that were significantly up- or downregulated in cell lines with a *CDH1* promoter methylation *vs* cell lines without promoter methylation (FDR<0.05). Table 2 shows the top 10 up- and downregulated genes based on the false discovery rate. Table 3 shows a subset of genes that are of particular interest because they are involved in the TGF*β* pathway, EMT control or cytoskeletal (re)organisation.

### Real-time PCR

**Figure 4:**
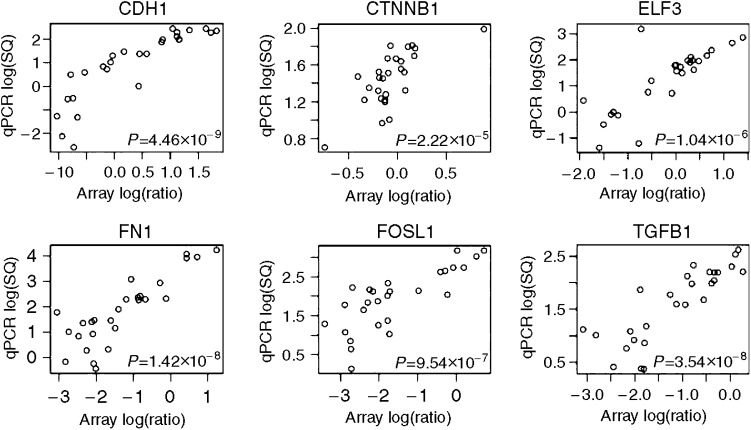
Correlation between cDNA microarray data and real-time qPCR for six genes.

Quantitative PCR was performed to validate cDNA microarray expression data for six differentially expressed genes (*CTNNB1, CDH1, ELF3, FN1, FOSL1* and *TGFB1*; see Table 4). The correlation between results from the microarray and qPCR was highly significant (Figure 4). The microarray expression data led us to hypothesise that breast cell lines with *CDH1* promoter methylation all have undergone EMT. To verify this, we also performed qPCR for genes that are involved in the regulation of *CDH1* expression (*TWIST1, ZFHX1B, SNAI1* and *SNAI2*) or (a marker for) EMT (*ILK, VIM* and *SERPINE1*), but could not be evaluated on the array because of poor hybridisation results or absence.

**Figure 5:**
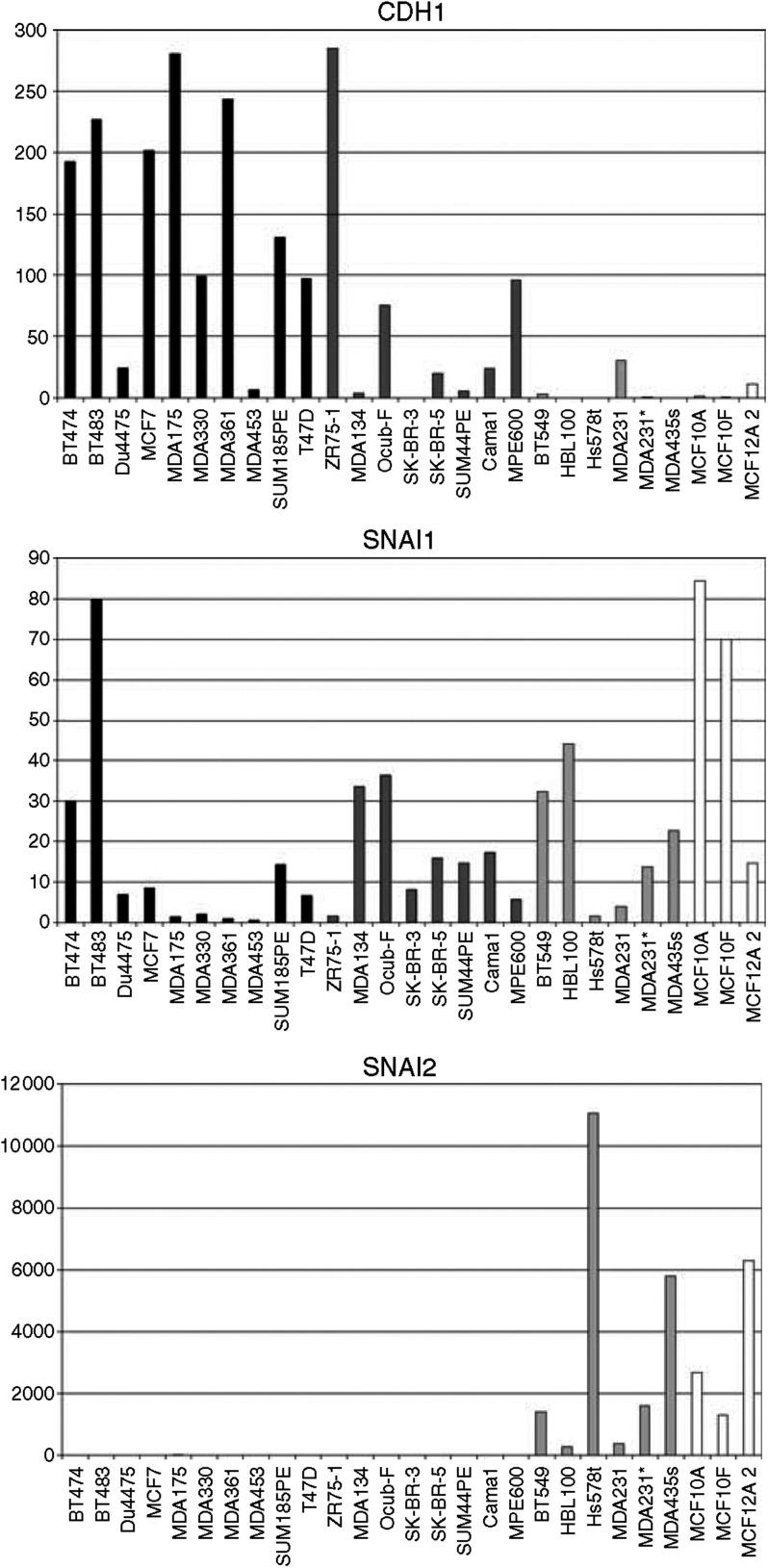
Three examples of qPCR data showing the following: *CDH1*, high expression in the ‘epithelial’ cluster; *SNAI1*, no significant difference; *SNAI2*, high expression in the ‘fibroblastic’ cluster. The *y*-axis represents the relative expression levels as determined by qPCR. Black bars=epithelial with wild-type *CDH1*, dark grey=epithelial, *CDH1* mutation, light grey=fibroblastic tumour, *CDH1* methylation, white=fibroblastic ‘normal’ lines, *CDH1* methylation.

As determined by one-way ANOVA, the normalised starting quantity, a measure for the amount of mRNA in the sample, differed significantly between tumour cell lines with *CDH1* promoter methylation with a fibroblastic morphology (‘Fibroblastic-Tumour’ cluster) and epithelial breast cancer cell lines (‘Epithelial-Ecad-Expressing’ and ‘Epithelial-CDH1-Mutated’ clusters) for several genes (Table 4). *CDH1* and *ELF3* were expressed at a significantly higher level in cells with an epithelial morphology, whereas *FN1, FOSL1, VIM, ZFHX1B, SNAI2, SERPINE1* and *TFGB1* showed a higher expression in fibroblastic breast tumour cells (Table 4 and Figure 5).

Cell lines in the ‘Epithelial-Ecad-Expressing’ subcluster showed considerable variation in *CDH1* expression, with higher expression levels predominantly in the ER^+^ cell lines. Remarkably, the highest levels of *ELF3* expression were identified in both SK-BR-3 and SK-BR-5 cell lines.

Several cell lines within the ‘Fibroblastic-Tumour’ subcluster show high expression of *TWIST1*, particularly BT549 and MDA-MB-435s, but for the whole group the results did not differ significantly from those of the epithelial cluster. Of the ‘Epithelial’ cluster, SUM44PE, which was originally derived from a lobular carcinoma (Ethier *et al*, 1993; van de Wetering *et al*, 2001), showed the highest expression. Remarkably, cell lines derived from normal breast epithelial cells, MCF10A, MCF10F and MCF12A, showed very high expression levels of *FN1, SERPINE1, ZFHX1B, SNAI2, TGFB1, TWIST1* and *VIM* and low values for *CDH1* and *ELF3*, which is comparable to expression data from cell lines in the ‘Fibroblastic-Tumour’ subcluster.

## DISCUSSION

Alterations in E-cadherin are an important event in carcinogenesis; however, there is controversy about the corollary of the type of E-cadherin inactivation (mutation or promoter hypermethylation) and the aggressiveness of tumour cells. In view of its function in adhesion, it is considered as an invasion suppressor, which is indeed corroborated by *in vitro* experiments (Vleminckx *et al*, 1991). Nevertheless, mutational inactivation is already identified in pre-invasive lobular carcinoma *in situ* (Vos *et al*, 1997), thereby supporting a role in early carcinogenesis instead of invasive capacity. In order to identify pathways that are affected by E-cadherin inactivation, we performed a genome-wide expression study on 27 human mammary cell lines, which are well characterised on E-cadherin RNA and protein expression status.

Cluster analysis of the microarray data identified two main clusters that coincide with the epithelial and fibroblastic phenotype of the cells, respectively. Importantly, the ‘Fibroblastic’ cluster included only cell lines with either partial or complete *CDH1* promoter methylation. This contrasts with the ‘Epithelial’ cluster that included cell lines with wild-type as well as cell lines with mutant *CDH1* status. Based on published data on *in vitro* invasion assays, cellular phenotype and gene expression profiles, Lacroix and Leclercq, (2004) classified breast cancer cell lines into three groups: luminal epithelial, weakly luminal epithelial and ‘mesenchymal’ or ‘stromal’ like. In our analyses, all cell lines belonging to both the luminal and weakly epithelial luminal type group into the ‘Epithelial’ cluster. The position of colorectal tumour cell lines as ‘epithelial’ subcluster rather than forming an out-group suggests that the origin of these cells is subordinate to the phenotype (epithelial or fibroblastic), underscoring the large phenotypic differences induced by EMT.

The fibroblastic phenotype of the cell lines in the latter cluster is strongly indicative of EMT. This is also supported by the increased invasiveness of these cell lines (BT549, Hs578T, MDA-MB-231 and MDA-MB-435s) *in vitro* and their metastatic potential in mouse models (Price *et al*, 1990; Thompson *et al*, 1992; Sommers *et al*, 1994a). Lacroix and Leclercq (2004) identified 72 differentially expressed genes between the (weakly) luminal epithelial and mesenchymal cell lines of which 15 genes (21%) coincide with differentially expressed genes in our ‘Epithelial’ *vs* ‘Fibroblastic’ cluster and are indicated in Tables 2, 3 and 4 with an asterisk. The finding that the three non-tumorigenic mammary cell lines derived from normal epithelium form a cluster close to the fibroblastic tumour cell lines is remarkable. However, given their fibroblastic morphology and the *CDH1* promoter methylation, this is not unexpected.

Our observation that EMT only occurs in breast cancer cell lines with CDH1 promoter hypermethylation and not with a CDH1 mutational inactivation questions the presumed central role of E-cadherin loss as the initial or primary cause for EMT. This is furthermore illustrated by the surprising lack of significantly differentially expressed genes when comparing cell lines with wild-type and mutant *CDH1.* It strongly suggests that E-cadherin transcriptional inactivation is an epi-phenomenon and part of an entire programme, with much more severe effects than loss of E-cadherin expression alone. The genes that are involved in this programme can be inferred from the significantly differentially expressed genes when comparing ‘Fibroblastic’ and ‘Epithelial’ cell lines. Two of the identified upregulated genes are upstream repressors of CDH1 transcription, thereby emphasising that E-cadherin itself is not the initiating event in this programme.

We identified 273 differentially expressed genes between breast cancer cell lines in the ‘Epithelial’ *vs* the ‘Fibroblastic’ cluster, underscoring that these two phenotypes are highly different (Lacroix and Leclercq, 2004). We hypothesise that mutational inactivation is selected for early in carcinogenesis and results in increased growth. In contrast, the transcriptional inactivation by promoter methylation seems part of a larger programme directed towards EMT, thereby increasing invasive and tumorigenic capacity or providing normal epithelial cells with the propensity to divide infinitely in culture as can be inferred from cluster 1B. The TGF*β* pathway and furthermore transcription factors that regulate E-cadherin (ZFHX1B and SNAI2), FOSL1 and other AP1/AP2 transcription factors, members of cytoskeleton organisation, IGFBPs, caveolae components, annexins and the AXL receptor tyrosine kinase seem part of such a programme. Further below we will discuss how the major groups of gene products that differentiate the ‘Fibroblastic’ and ‘Epithelial’ breast tumour cell line fit in the this EMT paradigm.

The increased expression of several genes involved in the TGF*β* pathway in the ‘Fibroblastic’ cluster, that is, *TGFβ1, TGFβ2* and their receptor *TGFβR2*, is in agreement with the important role of this pathway in the induction of EMT (Thiery, 2003 and references therein). Furthermore, one of the known downstream targets of the TGF*β* pathway is ZFHX1B/SIP1, which is a direct repressor of *CDH1* (Comijn *et al*, 2001). Another transcriptional repressor of *CDH1*, SNAI2/SLUG, a downstream target of the cKIT pathway (Perez-Losada *et al*, 2002), is also significantly upregulated in the ‘Fibroblastic’ cluster, suggesting that other pathways might also be involved in EMT-related E-cadherin downregulation. For two other transcription factors that are well known to regulate E-cadherin expression in relation to EMT, TWIST (Yang *et al*, 2004) and SNAI1 (Cano *et al*, 2000), no significantly altered expression was observed in ‘Fibroblastic’ cells. Together, this suggests that ZFHX1B and SNAI2 are the predominant transcriptional regulators of CDH1 accounting for the EMT phenotype of breast tumour cell lines. Remarkably, *TWIST* upregulation was reported in lobular breast cancer as an alternative for inactivating mutations of *CDH1* (Yang *et al*, 2004) and, moreover, SUM44PE, the only breast cancer cell line of lobular origin in our panel, showed the highest expression of *TWIST*. As *TWIST* is not significantly differentially expressed in the fibroblastic cell lines, we suggest that its protein product has a direct effect on *CDH1* and results in a similar phenotype as *CDH1* mutations, thereby contributing to the typical phenotype of lobular breast cancer. The lack of upregulation of SNAI1 is unexpected, especially given the recently identified role of this gene in breast cancer recurrence (Moody *et al*, 2005).

*FOSL1* (also called *FRA1*) and *FOSB*, albeit to a lesser extent, were significantly upregulated genes in cell lines with *CDH1* promoter methylation. These FOS family members form heterodimers with JUN family members (mainly, c-Jun, JunB and JunD) thereby forming the AP1 transcription factor (Karin *et al*, 1997). Various studies have shown that alterations of the composition of AP1 are related to changes in proliferation, malignant transformation and aggressiveness of cells (Mechta *et al*, 1997; Smith *et al*, 1999). Detectable FOSL1 protein expression in mammary carcinomas was demonstrated to be associated with poor differentiation, Ki67 and cyclin E expression and an oestrogen receptor-negative phenotype (Milde-Langosch *et al*, 2000). As an AP1 site has been identified in the *TGFBR2* promoter, upregulation of *FOSL1* and *FOSB* may stimulate *TGFBR2* transcription. Interestingly, RHOB binds to the promoter of *TGFBR2* and in this way prevents AP1-dependent transcription creating a negative feedback loop that regulates TGF*β* signal transduction (Adnane *et al*, 2002). *RHOB* was significantly downregulated in the ‘Fibroblastic’ cluster. Other targets of AP1 are extracellular matrix modulating enzymes that on their turn may contribute to an increased migratory and metastatic phenotype. Indeed, genes encoding metalloproteinases *MMP 2, 3, 14* and *15* as well as *PLAUR*, encoding the urokinase receptor, showed increased expression in the *CDH1* methylated cells.

Besides downregulation of E-cadherin and loss of cell–cell interactions, EMT is accompanied by extensive reorganisation of actin as well as intermediate filamental cytoskeleton. Therefore, it is not surprising that we observed a differential expression of various genes that encode parts of the intermediate filaments, including KRT7, -8, -13, -14, -19 and vimentin, a fibroblastic marker. Also genes that regulate the organisation and turnover of the F-actin filaments such as *RAC, MSN* as well as *RHOB* were differentially expressed (Table 2). The balance between *RHO* and *RAC* is shifted towards *RAC* in cell lines with fibroblastic morphology. The upregulation of *RAC* in these latter cells fits with increased protrusions and lamellopodia that are required for cell migration.

Also, annexin gene family members, of which *ANXA1, ANXA5* and *ANXA8* show increased expression in fibroblastic cells, may indirectly affect the cytoskeleton. AXL is a member of a family of receptor tyrosine kinases characterised by an extracellular domain resembling cell adhesion molecules and an intracellular conserved tyrosine kinase domain. Its upregulation in the ‘Fibroblastic-Tumour’ cluster is in agreement with the reported elevated expression in highly metastatic osteosarcoma cell lines (Nakano *et al*, 2003) and metastatic tumours including colon cancer, gastric cancer and melanoma (Quong *et al*, 1994; Craven *et al*, 1995; Wu *et al*, 2002).

We are aware that this study requires translation to tissue samples. Unfortunately, such a study is hampered by infiltrating lymphocytes that confound a reliable detection of CDH1 promoter hypermethylation by MSP (Lombaerts *et al*, 2004).

In conclusion, our data indicate that *CDH1* promoter hypermethylation but not *CDH1* mutational inactivation is a part of an entire EMT programme resulting in breast tumour cells with a more aggressive phenotype, thus enabling metastasis formation. At this moment, we do not know the initial steps for this epigenetically controlled EMT. Nevertheless, it has become generally accepted that metastasis is facilitated by EMT and thus interfering with this process in breast cancer might prevent tumour dissemination. Hence, targeting of abnormal TGF*β* signalling could be one of the main priorities in preventing EMT and its adverse effects on the prognosis of patients with breast cancer (Sokol *et al*, 2005). Future investigations will therefore be directed at verification of this transcriptional programme associated with *CDH1* methylation in primary breast tumour samples and an association with disease outcome.

## Figures and Tables

**Table 1:** Cell lines used in this study

**Cell line^(source)^**	**Tissue**	**Tumour type**	**Genotype confirmation**	**CDH1 promoter methylation**	**Protein expression**	**CDH1 mutation^(9)^**	**QPCR^a^**	**Flow index MESF**
BT474^(1)^	Breast	IDC	+	−	+		192.7	
BT483^(1)^	Breast	IDC	NIA	−			226.6	
BT549^(1)^	Breast	PIDC	NIA	±	−		3.2	
CAMA1^(1)^	Breast	C	+^(7)^	−	+	+	23.9	17
Du4475^(1)^	Breast	IDC	+	−	+	−	24.5	
HBL100^(1)^	Breast	Normal^(8)^	NIA^(8)^	+	−		0.0	
Hs578T^(1)^	Breast	CS	+	±	−	−	0.0	0
MCF10A^(1)^	Breast	Normal		±			1.0	
MCF10F^(1)^	Breast	Normal	+	±	−		0.3	
MCF12A^(1)^	Breast	Normal	+	±			10.7	
MCF7^(1)^	Breast	IDC	+	−	+	−	201.8	47
MDA-MB-134 VI^(1)^	Breast	IDC	+	−	−	+	3.8	
MDA-MB-175 VII^(1)^	Breast	IDC	+	−	+	−	281.1	
MDA-MB-231^(1)^	Breast	IDC	+	±	±	−	30.3	18
MDA-MB-231^*(6)^	Breast	IDC	^(6)^	±	−		0.3	
MDA-MB-330^(1)^	Breast	IDC	+	−	+	−	99.1	
MDA-MB-361^(1)^	Breast	AC	+	−	+	−	243.6	
MDA-MB-435s^(1)^	Breast	IDC	+	+	−		0.0	0
MDA-MB-453^(1)^	Breast	AC	+	−		−	6.8	
MPE600^(2)^	Breast	C	NIA	−	+	+	96.2	108
OCUB-F^(3)^	Breast	C	NIA	−	−	+	75.7	
SK-BR-3^(1)^	Breast	IDC	+	Del	−	+	0.1	0
SK-BR-5^(4)^	Breast	C	NIA	−	−	+	20.1	0
SUM185PE^(5)^	Breast		NIA	−	+		131.2	
SUM44PE^(5)^	Breast	ILC	NIA	−	−	+	5.4	
T47D^(1)^	Breast	IDC	+	−	+	−	97.2	121
ZR75-1^(1)^	Breast	IDC	+	−	+	−	284.8	103
LS180^(1)^	Colorectal	AC		−				
LS411N^(1)^	Colorectal	C		−				
SW480^(1)^	Colorectal	AC		−				
SW837^(1)^	Rectal	AC		−				

^(1)^American Tissue Type Collection (ATCC); ^(2)^Dr F Waldman; ^(3)^Riken Gene Bank; ^(4)^Dr E Stockert; ^(5)^Dr S Ethier; ^(6)^a clone spontaneously derived from MDA-MD-231 lacking allele 8 of TPOX; ^(7)^lost the 9.3 allele of THO1; ^(8)^HBL100 was originally isolated from a healthy female; however, it turned out to have a Y chromosome; ^(9)^after van de Wetering *et al*. (2001).

a Normalised real-time PCR SQ values for CDH1 expression; IDC=invasive ductal carcinoma; PIDC=papillary invasive ductal carcinoma; C=carcinoma; CS=carcinosarcoma; ILC=invasive lobular carcinoma; AC=adenocarcinoma; NIA=no information available; (blank)=not determined; Del=promoter deletion.

**Table 2:** Top 10 upregulated (>1) and downregulated (<1) genes in the ‘Fibroblastic-Tumour’ cluster when compared with breast tumour cell lines in the ‘Epithelial’ cluster

**Symbol**	**Name**	**Locus ID**	**Fold Change**	***P*-value**	**Keyword**
FOSL1	FOS-like antigen 1	8061	692	1.03E−07	Transcription factor
GBE1	Glucan (1,4-alpha-) branching enzyme 1	2632	9.77	6.09E−07	Sucrose metabolism
MMP15	Matrix metalloproteinase 15	4324	9.55	2.83E−06	Matrix degradation
AMPD1	Adenosine monophosphate deaminase 1	270	7.94	3.27E−06	Purine metabolism
COL4A2	Collagen type IV alpha 2	1284	36.3	1.31E−05	Matrix
ACADL	Acyl-coenzyme A dehydrogenase long chain	33	72.4	1.32E−05	Fatty acid metabolism
PLAUR	Plasminogen activator, urokinase receptor	5329	43.6	2.99E−05	Matrix degradation
AXL	AXL receptor tyrosine kinase	558	11.8	2.99E−05	
PHLDA1	Pleckstrin homology-like domain family A member 1	22822	13.2	3.56E−05	
ANXA5	Annexin A5	308	5.62	3.65E−05	
IGFBP2*	Insulin-like growth factor binding protein 2	3485	0.00371	1.27E−05	IGFBP
ST14*	Suppression of tumorigenicity 14	6768	0.0110	1.27E−05	Serine protease
TFDP2	Transcription factor Dp-2	7029	0.0355	2.58E−05	Transcription factor
XBP1	X-box binding protein 1	7494	0.0302	2.61E−05	Transcription factor
KRT13	Keratin 13	3860	0.0195	3.65E−05	Cytoskeleton
KIAA0089	KIAA0089	23171	0.170	7.60E−05	unknown
ELF3	E74-like factor 3	1999	0.0263	1.20E−04	Transcription factor
FXYD3	FXYD domain containing ion transport regulator 3	5349	0.0107	1.24E−04	Ion transport
KRT14	Keratin 14	3861	0.0195	1.24E−04	Cytoskeleton
IL1RN	Interleukin 1 receptor antagonist	3557	0.0933	1.24E−04	

* Genes also identified by Lacroix and Leclerq.

**Table 3:** Selection of differentially expressed genes involved in TGF*β*, matrix remodelling and cytoskeleton

**Symbol**	**Name**	**Locus ID**	**Fold change**	***P***	**Up or down**	**Group**
VEGFC	Vascular endothelial growth factor C	7424	30.9	2.42E−02	Up	Angiogenesis
PLOD2	Procollagen-lysine, 2-oxoglutarate 5-dioxygenase 2	5352	6.17	2.96E−02	Up	Angiogenesis
STC1	Stanniocalcin 1	6781	12.0	2.35E−03	Up	Angiogenesis
SPARC	Secreted protein, acidic, cysteine-rich	6678	28.2	2.57E−02	Up	AP1 target
CSF1*	Colony stimulating factor 1	1435	102	6.36E−05	Up	Cytokine
ITGB1	Integrin, beta 1	3688	5.13	2.03E−02	Up	Cytoskeleton
RAC2	Ras-related C3 botulinum toxin substrate 2	5880	6.76	1.59E−02	Up	Cytoskeleton
KRT7	Keratin 7	3855	0.0602	1.97E−02	Down	Cytoskeleton
KRT8	Keratin 8	3856	0.0251	5.38E−04	Down	Cytoskeleton
KRT19	Keratin 19	3880	0.00891	3.10E−04	Down	Cytoskeleton
RHOB	Ras homolog gene family, member B	388	0.0589	9.87E−03	Down	Cytoskeleton
ARHD	Ras homolog gene family, member D	29984	0.0871	3.42E−02	Down	Cytoskeleton
DSP*	Desmoplakin	1832	0.0631	1.22E−03	Down	Cytoskeleton/adhesion
CAV1	Caveolin 1	857	126	3.65E−05	Up	Cytoskeleton/adhesion
CAV2	Caveolin 2	858	10.7	1.50E−03	Up	Cytoskeleton/adhesion
CD44	CD44 antigen	960	51.3	5.27E−05	Up	Cytoskeleton/adhesion
FN1	Fibronectin 1	2335	17.4	2.36E−03	Up	Cytoskeleton/adhesion
MSN	Moesin	4478	115	1.26E−04	Up	Cytoskeleton/adhesion
S100A2	S100 calcium binding protein A2	6273	5.50	4.72E−02	Up	Cytoskeleton/adhesion
S100A3	S100 calcium binding protein A3	6274	7.41	1.08E−02	Up	Cytoskeleton/adhesion
CDH1*	E-cadherin	999	0.0589	2.82E−02	Down	Cytoskeleton/adhesion
MAPK1	Mitogen-activated protein kinase 1	5594	54.9	1.69E−03	Up	MAPK
COL5A1	Collagen type V alpha 1	1289	8.32	1.58E−02	Up	Matrix
COL15A1	Collagen type XV alpha 1	1306	3.31	3.94E−02	Up	Matrix
BMP1	Bone morphogenetic protein 1	649	9.55	1.85E−02	Up	Matrix degradation
MMP2	Matrix metalloproteinase 2	4313	27.5	9.29E−03	Up	Matrix degradation
MMP3	Matrix metalloproteinase 3	4314	2.45	4.81E−02	Up	Matrix degradation
MMP14*	Matrix metalloproteinase 14	4323	53.7	5.27E−05	Up	Matrix degradation
PLAU*	Plasminogen activator, urokinase	5328	14.45	3.32E−03	Up	Matrix degradation
SERPINE1*	Serine or cysteine proteinase inhibitor member 1	5054	19.1	1.44E−02	Up	Matrix degradation
SERPINE2	Serine or cysteine proteinase inhibitor member 2	5270	759	1.40E−04	Up	Matrix degradation
TPD52*	Tumor protein D52	7163	0.25	1.86E−02	Down	Morphogenesis
ANXA1	Annexin A1	301	6.31	3.82E−05	Up	Phospho-lipid binding
ANXA5	Annexin A5	244	5.75	3.66E−05	Up	Phospho-lipid binding
ANXA8	Annexin A8	244	3.09	3.24E−02	Up	Phospho-lipid binding
TGFB1	Transforming growth factor beta 1	7040	11.8	3.76E−02	Up	TGFbeta
TGFB2	Transforming growth factor beta 2	7042	23.4	1.50E−03	Up	TGFbeta
TGFBR2	Transforming growth factor beta receptor II	7048	11.2	9.56E−04	Up	TGFbeta
FST	Follistatin	10468	11.5	6.41E−04	Up	TGFbeta
FOSB	FBJ murine osteosarcoma viral oncogene homolog B	2354	2.24	4.47E−02	Up	Transcription factor
TFAP2A	Transcription factor AP-2 alpha	7020	0.224	2.29E−03	Down	Transcription factor
TFAP2C*	Transcription factor AP-2 gamma	7022	0.144	3.58E−02	Down	Transcription factor
MDM2*	Mouse double minute 2 homolog isoform	4193	0.380	5.59E−03	Down	Ubiquitination

Up indicates upregulated in the ‘fibroblastic’ cell lines.

* Genes also identified by Lacroix and Leclerq.

**Table 4:** Quantitative RT–PCR data

**Symbol**	**Name**	**Locus ID**	**Fold change**	***P*-value**	**Up or down**	**Keyword**
CPSF6	Cleavage and polyadenylation specific factor 6	11052	0.98	7.15E−01	NS	Housekeeping gene
HNRPM	Heterogeneous nuclear ribonucleoprotein M	4670	1.0	1.00E+00	NS	Housekeeping gene
TBP	TATA box binding protein	6908	1.0	1.00E+00	NS	Housekeeping gene
CTNNB1	Catenin beta 1	1499	1.1	1.00E+00	NS	Adhesion and transcription factor
SNAI1	Snail homolog 1	6515	1.3	1.00E+00	NS	Transcription repressor
TWIST1	Twist homolog 1	7291	1.7	1.00E+00	NS	Transcription repressor
ILK	Integrin-linked kinase	3611	1.26	9.50E−02	NS	TGFbeta
TFGB1	Transforming growth factor beta	7040	2.0	1.50E−02	Up	TGFbeta
SERPINE1	Serine or cysteine proteinase inhibitor	5054	5.1	3.00E−03	Up	Matrix degradation
FN1	Fibronectin 1	2335	2.8	2.00E−03	Up	Mesenchymal marker
FOSL1	FOS-like antigen 1	8061	2.4	0.00E+00	Up	Transcription factor
SNAI2*	Snail homolog 2, slug	6591	12	0.00E+00	Up	Transcription repressor
VIM*	Vimentin	7431	12	0.00E+00	Up	Mesenchymal marker
ZFHX1B	Zinc finger homeobox 1b, sip1	9839	8.9	0.00E+00	Up	Transcription repressor
CDH1	E-cadherin	999	0.15	0.00E+00	Down	Cytoskeleton/adhesion
ELF3	E74-like factor 3	1999	0.24	0.00E+00	Down	Transcription factor

Up indicates upregulated in the ‘fibroblastic’ cell lines. NS=not significant.

* Genes also identified by Lacroix and Leclerq.
